# Synthesis of Novel Peptides Using Unusual Amino Acids

**DOI:** 10.22037/ijpr.2020.113827.14509

**Published:** 2020

**Authors:** Bahareh Talaei, Vaezeh Fathi Vavsari, Saeed Balalaie, Armin Arabanian, Hamid Reza Bijanzadeh

**Affiliations:** a *Peptide Chemistry Research Institute, K. N. Toosi University of Technology, P.O. Box 15875-4416 Tehran, Iran. *; b *Medical Biology Research Center, Kermanshah University of Medical Sciences, Kermanshah, Iran.*; c *Department of Environmental Sciences, Faculty of Natural Resources and Marine Sciences, Tarbiat Modares University, Tehran, Iran.*

**Keywords:** Unusual amino acids, Synthesis of di-, tri-, and tetra- peptides, Baclofen, Gabapentin, Phenylalanine, Hydrogen bonding

## Abstract

Small peptides are valuable peptides due to their extended biological activities. Their activities could be categorized according to their low antigenicity, osmotic pressure, and also because of their astonishing bioactivities. For example, the aggression of Phe-Phe fibers via self-assembly and intermolecular hydrogen bonding is the main reason for the formation of Alzheimer’s β-amyloid fibrils. Hydrogen bonding is the main intramolecular interaction in peptides, while the presence of aromatic ring leads to the π-π stacking and affects the self-assembly and aggression. Thus, insertion of an unusual amino acid into peptide sequence facilitates the formation of intramolecular bonds, lipophilicity and its conformation. To design new small peptides with remarkable lipophilicity, it is an idea to employ *γ*-amino acid, such as gabapentin (H_2_N-Gpn-OMe) and baclofen (H_2_N-Baclofen-OMe), in the structure of small peptides to increase cell-penetrating properties and to prevent aggression of Phe-Phe fibrils in β-amyloids of Alzheimer’s disease. Some new tri- and tetrapeptides were synthesized through introducing biologically active gabapentin and baclofen to dipeptide of phenylalanine (Phe-Phe) through solution phase peptide synthesis strategy.

## Introduction

During digestion or degeneration of proteins, small peptides are formed that are of vital sources of nutrition for human and animals (1). Particularly, di- and tri-peptides are the most valuable ones due to the low antigenicity, osmotic pressure, and also because of their astonishing bioactivities, e.g. antioxidative, antimicrobial, antihypertension, and immunomodulatory (2, 3).

Beside the special biological activities of dipeptides, another surprising feature of them was discovered by Reches and Gazit in 2003. They found that *L*-Phe-*L*-Phe makes nanotubes in hexafluoro-2-propanol and water as solvents (4). Currently, the focus on the aggression of Phe-Phe fibers is because of their potential characteristic in the aggression of Alzheimer’s β-amyloid fibrils. It has been found that the π-π stacking among phenyl rings of –F19-F20- is the main reason of amyloids aggression (5-7). Therefore, finding the properties of such fibers opens doors to discover new methods of treating various nervous diseases. 

Hydrogen bonding is the main intramolecular interaction in peptides, while the presence of aromatic ring leads to the π-π stacking and affects the self-assembly and aggression. Thus, insertion of an unusual amino acid and sugars into peptide sequence increases the formation of intramolecular bonds, lipophilicity, and its conformation (8). Modification of the backbone of *α*-peptides can result in proteolytically stable sequences; one of the important properties in the design of analogues of biologically active sequences (9). Moreover, modification of peptides with glycosyl and other types of highly soluble amino acids moieties has gained prominence because of awesome functional characteristics of these biomolecules (10-12). For instance, glycoconjugation of lysine residues using sugar vinyl sulfoxide led to an antimicrobial compound that catalyzes digestion of Gram-negative *E*. *coli *cell wall (13). Recently, aspartic thioacid-containing peptides were synthesized and easily converted to *N*-glycopeptides through a chemoselective thioacid–glycosylamine ligation (14). Asparagine containing glycopeptides linked to various saccharides were also prepared to develop the synthetic method of such valuable compounds (15).

Gabapentin (Gpn) is an available antiepileptic drug which is also used as a medicine for neuropathic pains (16). Due to the presence of cyclohexyl group in Gpn structure, construction of peptides with Gpn residues could affect conformation and lipophilicity of the final peptide (17). Racemate baclofen is a lipophilic analogue of GABA which acts as a muscle relaxer and an antispastic agent (18). Existence of 4-chlorophenyl moiety in its structure increases its lipophilicity. Constructing peptides with highly lipophilicity may affect their permanently remaining into the central nervous system (19). In order to peptide design, Gpn may be employed as a stereochemically constrained equivalent of its parent unsubstituted γ-aminobutyric acid residue (20). Hence, designing new small peptides fibres with remarkable lipophilicity is a good idea to increase cell-penetrating properties and to prevent aggression of Phe-Phe fibrils in β-amyloids of Alzheimer’s disease. Alezra’s research team found that small peptides including *γ*-amino acids may act as turn inducer to either form stable structures or enhance bioactivity of the molecules (21-23). One of them is probably gelation that has been observed in active *γ*-peptides (24). Such properties have been found as new advantages of self-assembled peptides for medicine (25).

**Figure 1:**
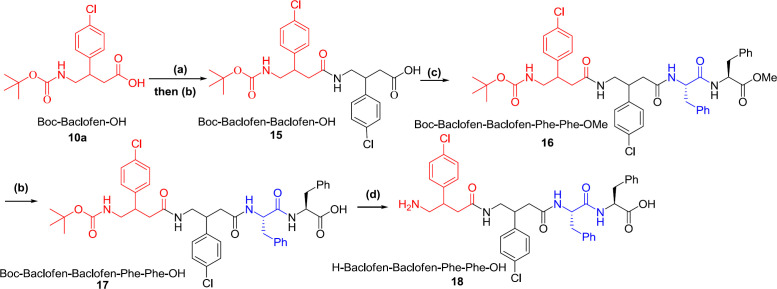
The structure of targeted small peptides

Due to increasing interest for the synthesis of low molecular weight peptides, primarily, we were encouraged to design and synthesis some novel small peptides; to value this desire, the target small peptides were designed to enrich with biologically active *γ*-amino acid (Gpn and baclofen) and Phe-Phe dipeptide in their backbones. Correspondingly, the following peptides were synthesized (Figure 1) H-Phe-Phe-Gpn-OH, H-Phe-Phe-Baclofen-OH, H-Baclofen-Phe-Phe-OH, H-Gpn-Phe-Phe-OH, and H-Baclofen-Baclofen-Phe-Phe-OH.

## Experimental

*General*

All solvents were purchased as reagent grade, dried, using standard conditions and stored over molecular sieves. NMR spectra were carried out on a Bruker Avance (DRX-300 or DR-X 500 MHz) spectrometer. Chemical shifts (δ) are reported in parts per million (ppm) relative to residual solvent as an internal reference. The following abbreviations were used to explain the multiplicities: s, singlet; *d*, doublet; *t*, triplet; *q*, quartet; *dd* doublet of doublets; *m*, multiplet; *br*, broad. The structure of all products was characterized by ^1^H NMR (300 MHz) and thin layer chromatography (TLC) on silica gel and used without further purification. The purified final compounds were fully characterized by IR spectroscopy, ^1^H NMR spectroscopy, and HR-mass spectrometry. Melting points were obtained on an Electrothermal* 9100* capillary melting point apparatus. High-resolution mass spectra (HRMS) were performed on an Apex Qe-FTICR mass spectrometer. The IR spectra were obtained on a FT-IR ABB (FTLA 2000) spectrometer in liquid film and KBr pellets.

*General procedure for the synthesis of di-, tripeptide with protected C-Terminal and N-Terminal* *(Peptide Coupling):*

Boc-AA-OH (1 mmol), TBTU (1.1 mmol), HOBt.H_2_O and ethyl acetate (7 mL) were stirred for 10 min. Then, H-AA-OMe (1.2 mmol) and diisopropylethylamine (DIPEA) (3 mmol) were added and the mixture was stirred for 12 h. The progress of reaction was monitored by TLC (H_2_O/Methanol/ethyl acetate 1:2:10). The product was taken in ethyl acetate (60 mL). The aqueous phase was extracted with ethyl acetate and this operation was done repeatedly. The organic layer was washed with Na_2_CO_3_ 3% (3 × 50 mL), brine (2 × 50 mL), and acidified with a dilute solution of citric acid 20 % (3 × 50 mL), and brine (2 × 50 mL), then dried over anhydrous sodium sulfate and filtered and concentrated by rotary evaporator to get the product.

*General procedure for deprotection* *of* *C-Terminus*

To Boc-AA-AA-OMe (1 mmol), a mixture of MeOH (25 mL) and NaOH (4 mL, 2 M) was added and the progress of saponification was monitored by thin layer chromatography (TLC). The reaction mixture was stirred. After 10h, MeOH was evaporated under vacuum, the residue was taken in EtOAc (50 mL), washed with water (2 × 50 mL); acidity of the aqueous layer was adjusted at pH = 2 using citric acid 20% and it was extracted with EtOAc (3 × 50 mL). The extracts were pooled, dried over anhydrous Na_2_SO_4_ and evaporated *in vacuum*. 

*General procedure for final Deprotection*

To *N*-protected compound (1 mmol), triethylsilan (3 mmol) and a mixture of anhydrous TFA and DCM (1:1 v/v) was gradually added and stirred well. The progress of reaction was monitored by TLC (H_2_O/MeOH/EtOAc 1:2:10). Then, excess solvent was evaporated under reduced pressure. The residue was purified by dissolving it in CH_2_Cl_2_ and recrystallized by adding Et_2_O. Then, the product was collected on a filter, washed with Et_2_O, and dried at 50 °C *in* *vacuum*.

And since zwitterions have minimal solubility at their isoelectric point, final product was isolated by precipitating it from water by adjusting the pH to its particular isoelectric point. Then, product was collected on a filter, and dried at 50 ˚C *in vacuum*.

*Boc-Phe-OH* ***1*** **:**

mp: 88-88.7 °C; IR ( KBr , cm^-1^ ) : 3435, 3372, 3034, 2983, 1689, 1653; ^1^H-NMR ( 300 MHz, CDCl_3 _) mixture of two isomers ( 60 : 40 ): *δ* = 10.57 ( *brs*, 1H, -COOH ); 7.18-7.33 ( *m*, 5H, H-Ar ) mixture of two isomers; 6.56-6.58 ( *d*, 1H, *J *= 7.3 Hz, Phe NH ) minor; 5.13-5.16 ( *d*, 1H, *J *= 7.9 Hz, Phe NH ) major; 4.61-4.67 ( *m*, 1H, C^α^H of Phe ) major; 4.09-4.16 ( *m*, 1H, C^α^H of Phe) minor; 3.18-3.24 ( *m*, 2H, Phe diastereotopic C^β^H ) mixture of two isomers; 3.04-3.11 ( *m*, 1H, Phe diastereotopic C^β^H ) major; 2.91-2.94 ( *m*, 1H, Phe diastereotopic C^β^H ) minor; 1.42 ( *s*, 9H, Boc-CH_3_ ) major; 1.32 ( *s*, 9H, Boc-CH_3_) minor; ppm; ^13^C-NMR ( 75 MHz, CDCl_3_) mixture of two stereoisomers: *δ* = 177.0 ( C of COOH ) minor; 176.1 ( C of COOH ) major; 156.7 ( C of NCOO ) minor; 155.4 ( C of NCOO ) minor; 136.5 ( C_ipso_ of phenyl ring attached to CH_2_ ) minor; 136.0 ( C_ipso_ of phenyl ring attached to CH_2 _) major; 129.4 ( *m*-C^’^s of phenyl ring ) mixture of two isomers; 128.5 ( *o*-C^’^s of phenyl ring ) mixture of two isomers; 127.0 ( *p*-C of phenyl ring ) mixture of two isomers; 81.6 ( C of Boc ) minor; 80.2 ( C of Boc ) major; 56.2 ( *α*-C of Phe ) minor; 54.3 ( *α*-C of Phe ) major; 38.9 ( *β*-C of Phe ) minor; 37.8 ( *β*-C of Phe ) major; 28.3 ( C of Boc-CH_3 _) major; 28.0 ( C of Boc-CH_3 _) minor; ppm. 

*Boc-Phe-Phe-OMe* ***2***

mp: 122-124 °C; IR (KBr, cm^-1^) : 3332, 3029, 1745, 1680, 1658; ^1^H-NMR (500 MHz, DMSO-*d*_6__)_ *δ *= 8.31 (*d*, 1H, *J *= 7.6 Hz, Phe (2) NH*)*; 7.16-7.29 (*m*, 10H, H-Ar*)*; 6.84 (*d*, 1H, *J *= 8.8 Hz, Phe (1) NH*)*; 4.49-4.51 (*m*, 1H, C^α^H of Phe (2)*)*; 4.17-4.18 (*m*, 1H, C^α^H of Phe (1)); 3.57 (*s*, 3H, -OCH_3_*)*; 3.02-3.03 (*m*, 1H, Phe (2) diastereotopic C^β^H*)*; 2.95-2.98 (*m*, 1H, Phe (2) diastereotopic C^β^H*)*; 2.86-2.88 (*m*, 1H, Phe (1) diastereotopic C^β^H*)*; 2.66-2.68 (*m*, 1H, Phe (1) diastereotopic C^β^H*)*; 1.27 (*s*, 9H, Boc-CH_3_*)* ppm; ^13^C-NMR (125 MHz, DMSO-*d*_6__)_ *δ* = 172.1 (C of -COOMe); 171.7 (C of -CON); 155.0 (C of NCOO); 137.2 (C_ipso_ of phenyl ring attached to CH_2_); 136.9 (C_ipso_ of phenyl ring attached to CH_2_); 129.1,129.0 (*m*-C^’^s of phenyl rings); 128.2, 127.9 (*o*-C^’^s of phenyl rings); 126.5, 126.1 (*p*-C of phenyl rings); 78.0 (C of Boc); 55.4 (-CH); 53.5 (-CH); 51.7 (-OMe); 37.4 (-CH_2_); 36.7 (-CH_2_); 28.0 (Boc-CH_3_) ppm.HRMS-ESI: Calcd. for C_24_H_31_N_2_O_5_ [M+H]^+^:427.2233; found: 427.2232. Calcd. for C_24_H_30_N_2_NaO_5_ [M+Na]^+^:449.2052; found: 449.2051. Calcd. for C_24_H_30_KN_2_O_5_ [M+K]^+^:465.1793; found: 465.1792. Calcd. for C_48_H_60_NaN_4_O_10_ [2M+Na]^+^: 875.4209; found: 875.4209.

*Boc-Phe-Phe-OH* ***3***

mp: 133-135 °C; IR (KBr, cm^-1^) : 3429, 3291, 1680, 1663; ^1^H-NMR (300 MHz, CDCl_3)_ *δ *= 7.05-7.26 (*m*, 10H, H-Ar*)*; 6.81 (*brs*, 1H, Phe (2) NH*)*; 5.16 (*brs*, 1H, Phe (1) NH*)*; 4.68(*brs*,1H, C^α^H of Phe (2); 4. 37 (*brs, *1H, C^α^H of Phe (1); 3.13-3.16 (*m*, 1H, Phe diastereotopic C^β^H*)*; 2.90-3.02 (*m*, 3H, Phe diastereotopic C^β^H*)*; 1.33(*s*, 9H, Boc-CH_3_*)* ppm; ^13^C-NMR (75 MHz, CDCl_3)_ *δ* = 175.1 (C of –COOH); 171.6 (C of –CON); 155.7 (C of NCOO); 136.4, 136.3 (C_ipso_ of phenyl ring attached to CH_2_); 129.4 (*m*-C^’^s of phenyl rings); 128.6, 128.5 (*o*-C^’^s of phenyl rings); 127.1, 126.9 (*p*-C of phenyl rings); 80.5 (C of Boc); 55.6 (*α*-C of Phe); 53.9 (*α*-C of Phe); 38.0 (*β*-C of Phe); 37.4 (*β*-C of Phe); 28.19 (C of Boc-CH_3)_ ppm. HRMS-ESI: Calcd. for C_23_H_28_N_2_NaO_5_ [M+Na]^+^:435.1895; found: 435.1894. Calcd. for C_46_H_56_N_4_NaO_10_ [2M+Na]^+^: 847.3893; found: 847.3893.

*Boc-Phe-Phe-Gpn-OMe* ***4***

mp: 107-108 °C; IR (KBr, cm^-1^) :3337, 2926, 1740, 1705, 1695, 1642; ^1^H-NMR (500 MHz, DMSO-*d*_6__)_ *δ* = 8.01 (*d*, 1H, *J *= 8.3 Hz, Phe (2) NH*)*; 7.74-7.75 (*brt*, 1H,* J* = 5.8 Hz, Gpn NH*)*; 7.05-7.24 (*m*, 10H, H-Ar); 6.87-6.89 (*d*, 1H, *J *=8.6 Hz, Phe(1) NH*)*; 4.61-4.63 (*m*, 1H, C^α^H of Phe(2)*)*; 4.1-4.11 (*m*, 1H, C^α^H of Phe(1)*)*; 3.54 (*s*, 3H, -OCH_3_*)*; 3.18-3.22 (*dd*, 1H,* J* = 13.3, 6.5 Hz, C^γ^H of Gpn*)*; 3.05-3.08 (*m*, 1H, C^γ^H of Gpn*)*; 2.92-2.96 (*dd*, 1H, *J* = 13.5, 5.8 Hz, Phe (2) diastereotopic C^β^H*)*; 2.81-2.85 (2*d*, 2H, *J* = 13.5 Hz, Phe (2) diastereotopic C^β^H*,* and Phe (1) diastereotopic C^β^H*)*; 2.63-2.65 (*m*, 1H, Phe (1) diastereotopic C^β^H*)*; 2.18 (*s*, 2H, C^α^H of Gpn*)*; 1.26 (*s*, 9H, Boc-CH_3_*)*; 1.12-1.40 (*m*, 10H, cyclohexyl ring protons*)* ppm; ^13^C-NMR (125 MHz, DMSO-*d*_6__)_ *δ* = 171.7 (C of COOMe); 171.2 (C of NCO); 171.0 (C of NCO); 155.0 (C of NCOO); 138.0, 137.5 (C_ipso_ of phenyl ring attached to CH_2)_; 129.2, 129.0 (*m*-C^’^s of phenyl rings); 128.0 (*o*-C^’^s of phenyl rings); 126.2, 126.0 (*p*-C of phenyl rings); 78.0 (C of Boc); 55.8 (*α*-C of Phe); 53.7 (*α*-C of Phe); 50.9 (C of -OCH_3)_; 44.3 (C of CH_2_NH_2)_; 39.0 (*β*-C of Gpn); 38.0 (*β*-C of Phe); 37.5 (*β*-C of Phe);36.7 (C of CH_2_COO); 32.4 (*γ*-C’s of Gpn); 28.0 (C of Boc-CH_3)_; 25.3 *ω*-C of Gpn); 20.9 (*δ*-C’s of Gpn) ppm. HRMS-ESI: Calcd. for C_33_H_46_N_3_O_6_ [M+H]^+^:580.3388; found: 580.3387. Calcd. for C_33_H_45_N_3_NaO_6_ [M+Na]^+^: 602.3209; found: 602.3208. Calcd. For C_33_H_45_KN_3_O_6 _[M+K]^+^:618.2950; found: 618.2948.

*Boc-Phe-Phe-Gpn-OH* ***5***

mp: 98-100 °C; IR (KBr, cm^-1^) : 3316, 2963, 2921, 1724, 1663.^ 1^H-NMR (500 MHz, DMSO-*d*_6__)_ *δ* = 12.64 (*br*, 1H, COOH); 8.08 (*d*, 1H, *J *= 7.5 Hz, Phe (2) NH); 7.42 (*brs*, 1H, Gpn NH); 7.16-7.28 (*m*, 10H, H-Ar); 6.82 (*d*, 1H, *J *= 8.7 Hz, Phe (1) NH); 4.46-4.47 (*m*, 1H, C^α^H of Phe (2); 4.16 (*m*, 1H, C^α^H of Phe (1); 3.05-3.09 (*m*, 1H, C^γ^H of Gpn); 2.64-2.96 (*m*, 5H, C^γ^H of Gpn, Phe diastereotopic C^β^H); 1.96 (*s*, 2H, C^𝜶^H of Gpn); 1.27 (*s*, 9H, Boc-CH_3)_; 1.12-1.40 (*m*, 10H, cyclohexyl ring protons) ppm. ^ 13^C-NMR (125 MHz, DMSO-*d*_6__)_ *δ* = 175.8 (C of COOH); 172.7 (C of NCO); 171.6 (C of NCO); 155.1 (C of NCOO); 138.1, 137.3 (C_ipso_ of phenyl ring attached to CH_2_); 129.2, 129.1 (*m*-C^’^s of phenyl rings); 128.2, 127.9 (*o*-C^’^s of phenyl rings); 126.4, 126.1 (*p*-C of phenyl rings); 78.0 (C of Boc); 55.6 (*α*-C of Phe); 53.3 (*α*-C of Phe); 43.0 (C of CH_2_NH_2)_; 39.8 (*β*-C of Gpn); 38.7 (C of CH_2_COO);37.4 (*β*-C of Phe); 36.8 (*β*-C of Phe); 36.2 (*γ*-C’s of Gpn); 28.1 (C of Boc-CH_3)_; 25.4 (*ω*-C of Gpn); 22.4 (*δ*-C’s of Gpn) ppm. ESI-Neg-HRMS: Calcd. for C_32_H_42_N_3_O_6_ [M-H]^+^:564.3094; found: 564.3092.

*H-Phe-Phe-Gpn-OH* ***6***

mp: 285-286 °C (dec.); IR (KBr, cm^-1^): 3260, 3061, 2931, 1683, 1560. ESI-MS: Calcd. for C_27_H_34_N_3_O_4_ [M-1]^+^: 464.2563; found: 464.2562. 

*Boc-Phe-Phe-Baclofen-OMe* ***7*** *:*

mp: 178-180 °C; IR (KBr, cm^-1^): 3368, 3338, 2945, 1730, 1689, 1651. ^1^H-NMR (300 MHz, DMSO-*d*_6__)_ *δ* = 7.90-8.03 (*m*, 2H, Baclofen NH and Phe (2) NH); 7.17-7.31 (*m*, 14H, H-Ar); 6.84-6.87 (*d*, 1H, *J* = 8.1 Hz, Phe(1) NH); 4.43-4.45 (*m*, 1H, C^α^H of Phe (2); 4.02-4.09 (*m*, 1H, C^α^H of Phe(1)); 3.45 (*s*, 3H, -OCH_3)_; 3.1-3.2 (m, 3H, C^β^H of Baclofen and C^γ^H of Baclofen); 2.78-2.82 (*m*, 2H, Phe diastereotopic C^β^H); 2.53-2.72 (*m*, 4H, Phe diastereotopic C^β^H and Baclofen diastereotopic *C*^α^*H*); 1.27 (*s*, 9H, Boc-CH_3)_ ppm. ^13^C-NMR (75 MHz, DMSO-*d*_6__)_ *δ* = 171.7 (C of COOMe); 171.2 (C of NCO); 170.7 (C of NCO); 156.0 (C of NCOO); 140.8 (C_ipso_-Cl); 138.0, 137.5 (C_ipso_ of Phe phenyl ring attached to CH_2)_; 131.1 (C_ipso_ of Baclofen phenyl ring attached to CH_2)_; 129.4, 129.1 (m-C^’^s of phenyl rings); 128.1,12127.9 (*o*-C^’^s of phenyl rings); 126.2, 126.0 (*p*-C of phenyl rings); 78.1 (C of Boc); 55.8 (*α* -C of Phe); 53.7 (*α*-C of Phe); 51.2 (C of -OCH_3)_; 43.6 (*β* -C of Baclofen); 41.0 (*β*-C of Phe); 37.9 (*β*-C of Phe); 37.6 (*γ *-C of Baclofen); 37.5 (*α* -C of Baclofen); 28.1 (C of Boc-CH_3)_ ppm. HRMS-ESI: Calcd. for C_34_H_41_ClN_3_O_6_ [M+H]^+^:622.2687; found: 622.2686. Calcd. for C_34_H_40_ClN_3_NaO_6_ [M+Na]^+^:644.2506; found: 644.2505. Calcd. For C_34_H_40_ClKN_3_O_6_ [M+K]^+ ^:660.2246; found: 660.2245.

*Boc-Phe-Phe-Baclofen-OH* ***8***

mp: 135-136 °C; IR (KBr, cm^-1^): 3338, 2929, 1729, 1689, 1651. ^1^H-NMR (300 MHz, DMSO-*d*_6__)_ *δ* = 12.02 (*brs*, 1H, -COOH); 7.94-8.04 (*m*, 2H, Baclofen NH, Phe (2) NH); 7.17-7.32 (*m*, 14H, H-Ar); 6.86-6.88 (*m*, 1H, Phe(1) NH); 4.43 (*m*, 1H, C^α^H of Phe (2); 4.09 (*m*, 1H, C^α^H of Phe(1)); 3.45 (*m*, 1H, C^β^H of Baclofen); 3.16 (*brs*, 2H, C^γ^H of Baclofen); 2.48-2.78 (*m*, 6H, Phe diastereotopic C^β^H, Baclofen diastereotopic C^α^H); 1.27 (*s*, 9H, Boc-CH_3)_ ppm. ^13^C-NMR (75 MHz, DMSO-*d*_6__)_* δ *= 172.8 (C of COOH); 171.7 (C of NCO); 171.2 (C of NCO); 155.1 (C of NCOO); 140.8 (C_ipso_- Cl); 138.0, 137.4 (C_ipso_ of Phe phenyl ring attached to CH_2_); 131.2 (C_ipso_ of Baclofen phenyl ring attached to CH_2)_; 129.7, 129.6, 129.2 (*m*-C^’^s of phenyl rings); 128.1, 128.0, 127.9 (*o*-C^’^s of phenyl rings); 126.2, 126.1 (*p*-C of phenyl rings); 78.1 (C of Boc); 55.8 (*α*-C of Phe); 53.7 (*α*-C of Phe); 43.6 (*β*-C of Baclofen); 40.8 (*β*-C of Phe); 37.9 (*β*-C of Phe); 37.5 (*γ*-C of Baclofen); 37.0 (*α*-C of Baclofen); 28.1 (C of Boc-CH_3)_ ppm. HRMS-ESI: Calcd. for C_33_H_39_ClN_3_O_6_ [M+H]^+^: 608.2528; found: 608.2527. Calcd. for C_33_H_38_ClN_3_NaO_6_[M+Na]^+^: 630.2347; found: 630.2346.

*H-Phe-Phe-Baclofen-OH* ***9***

mp: 85-86 °C; IR (KBr, cm^-1^): 3300, 3089, 2928, 1700, 1675, 1652. ^1^H-NMR (300 MHz, DMSO-*d*_6__)_ *δ* = 8.42 (*d*, 1H, *J *= 7.5 Hz Phe (2) NH); 8.07-8.17 (*m*, 1H, Baclofen NH); 7.07-7.36 (*m*, 14H, H-Ar); 4.42-4.46 (*m*, 1H, C^α^H of Phe (2); 3.60-3.70 (*m*, 1H, C^α^H of Phe (1); 3.24-3.28 (*m*, 1H, C^β^H of Baclofen); 3.16-3.24 (*m*, 2H, C^γ^H of Baclofen); 2.64-2.95 (*m*, 4H, Phe diastereotopic C^β^H); 2.40-2.60 (*m*, 2H, Baclofen diastereotopic C^α^H) ppm. ^13^C-NMR (75 MHz, DMSO-*d*_6__)_ *δ* = 171.3, 173.0 (C of COOH); 170.8, 170.7, 170.6 (C of NCO); 141.3, 141.2,137.5, 136.7, 136.6, 131.1, 129.6, 129.4, 129.1, 128.3, 128.1, 126.6, 126.4 (C-Ar.); 54.6 (*α*-C of Phe); 53.9 (*α*-C of Phe); 44.6 (*β*-C of Baclofen); 44.0, 43.9 (*β*-C of Phe); 40.8 (*γ*-C of Baclofen); 37.8 (*α*-C of Baclofen) ppm. HRMS-ESI: Calcd. for C_28_H_31_ClN_3_O_4_ [M+H]^+^:508.2002; found: 508.2001. Calcd. for C_28_H_30_ClN_3_NaO_4 _[M+Na]^+^: 530.1824; found: 530.1823. Calcd. for C_28_H_30_ClKN_3_O_4_ [M+K]^+^: 546.1566; found: 546.1565.

*Boc-Baclofen-OH* ***10a***

mp: 156-157 °C; IR (KBr, cm^-1^): 3301, 1699, 1637. ^1^H-NMR ( 300 MHz, DMSO-*d*_6_ ) *δ *= 12.05 ( *s*, 1H, OH ); 7.32 ( *d*, 2H, *J *= 8.3 Hz, Baclofen *m*-H’s of phenyl ring ); 7.22 ( *d*, 2H, *J *= 8.47 Hz, Baclofen *o*-H’s of phenyl ring ); 6.83-6.87 ( *t*, 1H, *J *= 5.4 Hz, NH ); 3.10-3.20 ( *m*, 1H, C^β^H of Baclofen ); 3.06 ( *t*, 2H, *J* = 5.7 Hz, C^γ^H of Baclofen ); 2.60-2.70 ( *dd*, 1H, *J *= 15.9, 4.9 Hz, C^α^H of Baclofen ); 2.41-2.49 ( *dd*, 1H, *J *= 15.9, 9.5 Hz, C^α^H of Baclofen ); 1.31 ( *s*, 9H, Boc-CH_3 _) ppm. ^13^C-NMR ( 75 MHz, DMSO-*d*_6_ ) *δ* = 172.9 ( C of COOH ); 155.5 ( C of NCOO ); 141.2 ( C_ipso_-Cl ); 130.9 ( C_ipso_ of phenyl ring attached to CH_2_ ); 129.7 ( *m*-C^’^s of phenyl ring ); 128.0 ( *o*-C^’^s of phenyl ring ); 77.5 ( C of Boc ); 45.1 ( *β*-C of Baclofen ); 41.3 ( C of CH_2_NH_2_ ); 37.6 ( C of CH_2_COO ); 28.1 ( C of Boc-CH_3 _) ppm. 

*Boc-Gpn-OH* ***10b***

mp: 127-128 °C; IR (KBr, cm^-1^) : 3413, 3085, 2931, 1714, 1668; ^1^H-NMR ( 300 MHz, CDCl_3 _) *δ* = 10.10 ( *brs*, 1H, COOH ); 5.01-5.04 ( *t*, 1H, *J* = 6.8 Hz, NH ); 3.14-3.16 ( *d*, 2H, *J *= 6.8 Hz, CH_2_NH_2_ ); 2.31 ( *s*, 2H, CH_2_COO ); 1.42 ( *s*, 9H, Boc-CH_3_ ); 1.22-1.49 ( *m*, 10H, cyclohexyl ring prptons ) ppm; ^13^C-NMR ( 75 MHz, CDCl_3 _) *δ* = 175.6 ( C of COOH ); 157.5 ( C of NCOO ); 80.2 ( C of Boc ); 47.3 ( C of CH_2_NH_2 _); 40.9 ( *β*-C of Gpn ); 37.6 ( C of CH_2_COO ); 33.9 ( *γ*-C’s of Gpn ); 28.3 ( C of Boc-CH_3 _); 25.8 ( *ω*-C of Gpn ); 21.3 ( *δ*-C’s of Gpn ) ppm.

*Boc-Baclofen-Phe-OMe* ***11a***

mp: 150-151 °C; IR (KBr, cm^-1^): 3370, 3361, 2973, 1738, 1700, 1685, 1646. ^1^H-NMR (300 MHz, CDCl_3)_ *δ* = 6.87-7.31 (*m*, 9H, Phenyl rings protons); 6.52, 6.26 (*m*, 1H, Phe NH); 4.75-4.81 (*m*, 1H, C^α^H of Phe); 4.53-4.56 (*m*, 1H, Baclofen NH); 3.71, 3.67 (*s*, 3H, -OCH_3)_; 3.22-3.46 (*m*, 3H, C^β^H of Baclofen, C^γ^H of Baclofen); 3.04-3.10 (*m*, 1H, Phe diastereotopic C^β^H); 2.98-3.0 (*m*, 1H, Phe diastereotopic C^β^H); 2.53-2.63 (*m*, 1H, Baclofen diastereotopic C^α^H); 2.30-2.43 (*m*, 1H, Baclofen diastereotopic C^α^H); 1.41 (*s*, 9H, Boc-CH_3)_ ppm. ^13^C-NMR (75 MHz, CDCl_3)_ *δ* = 171.9, 171.8 (C of NCO); 170.7, 170.4 (C of COOMe); 156.3 (C of NCOO); 140.1, 139.9 (C_ipso_-Cl); 135.9, 135.8 (C_ipso_ of Baclofen, phenyl ring attached to CH_2_); 132.8, 132.7 (C_ipso_ of Phe, phenyl ring attached to CH_2_); 129.2, 129.1, 129.0, `128.9, 128.8, 128.5 (*o*-C^’^s *&* *m*-C^’^s of phenyl rings); 127.0 (*p*-C of phenyl ring); 79.6 (C of Boc); 53.3, 53.2 (*α*-C of Phe); 52.3, 52.2 (C of -OCH_3)_; 45.1 (*β*-C of Baclofen); 42.4, 42.0 (*γ*-C of Baclofen), 39.9 (*β*-C of Phe); 37.7, 37.6 (*α*-C of Baclofen); 28.3 (C of Boc-CH_3)_ ppm. HRMS-ESI: Calcd. for C_25_H_31_ClN_2_O_5_ [M+H]^+^: 475.1998; found: 475.1997. Calcd. for C_25_H_31_ClN_2_NaO_5_ [M+Na]^+^:497.1817; found: 497.1816. Calcd. For C_25_H_31_ClKN_2_O_5_[M+K]^+^: 513.1557; found: 513.1556.

*Boc-Gpn-Phe-OMe* ***11b***

mp: 135-136 °C; IR (KBr, cm^-1^): 3200-3400, 1740, 1689, 1658. ^1^H-NMR (300 MHz, CDCl_3)_ *δ* = 7.72 (*d*, 1H, *J *= 7.4 Hz, Phe NH); 7.13-7.24 (*m*, 5H, H-Ar); 5.24 (*brt*, 1H, Gpn NH); 4.75-4.80 (*m*, 1H, C^α^H of Phe); 3.66 (*s*, 3H, -OCH_3)_; 3.12-3.22 (*m*, 2H, C^γ^H of Gpn); 2.87-2.98 (*m*, 2H, Phe diastereotopic C^β^H); 2.03 (*s*, 2H, C^α^H of Gpn); 1.4 (*s*, 9H, Boc-CH_3)_; 0.8-1.37 (*m*, 10H, cyclohexyl ring protons) ppm. ^13^C-NMR (75 MHz, CDCl_3)_ *δ*= 172.5 (C of COOMe); 171.4 (C of NCO); 157.2 (C of NCOO); 136.6 (C_ipso_ of phenyl ring attached to CH_2_); 129.2, 129.1 (*m*-C^’^s of phenyl ring); 128.4, 128.3 (*o*-C^’^s of phenyl ring); 126.8 (*p*-C of phenyl ring); 79.2 (C of Boc); 53.7 (*α*-C of Phe); 52.3 (C of -OCH_3)_; 46.8, 42.2, 38.0, 37.3, 34.3, 33.7; 28.3 (C of Boc-CH_3)_; 27.8, 25.9, 21.4 ppm.

*Boc-Baclofen-Phe-OH* ***12a***

mp: 145-146 °C; IR (KBr, cm^-1^): 3419, 3358, 2981, 2931, 1717, 1686. ^1^H-NMR (300 MHz, DMSO-*d*_6__)_ *δ *= 12.65 (*brs*, 1H, -COOH); 8.12-8.17 (*t*, 1H, *J* = 7.5 Hz, Phe NH); 6.97-7.26 (*m*, 9H, H-Ar); 6.72-6.83 (2*t*, 1H, *J* = 5.5 Hz, Baclofen NH); 4.25-4.34 (*m*, 1H, C^α^H of Phe); 2.73-3.15 (*m*, 5H, C^β^H of Baclofen, C^γ^H of Baclofen, Phe diastereotopic C^β^H); 2.28-2.44 (*m*, 2H, Baclofen diastereotopic C^α^H); 1.41 (*s*, 9H, Boc-CH_3)_ ppm. ^13^C-NMR (75 MHz, DMSO-*d*_6_) Mixture of two diasteromers :* δ* = 172.9 (C of COOH); 170.5, 170.3 (C of NCOO); 155.5 (C of NCOO); 141.3, 141.1, 137.7, 137.3, 130.8, 130.7, 129.5, 129.0, 128.9, 128.1, 128.0, 127.9, 126.3, 126.2 (C-Ar); 77.5 (C of Boc); 53.3 (*α*-C of Phe); 44.8 (*β* -C of Baclofen); 41.5 (*β*-C of Phe); 41.2 (*γ* -C of Baclofen); 36.6, 36.7 (*α*-C of Baclofen); 28.1 (C of Boc-CH_3)_ ppm. ESI-MS: Calcd. for C_24_H_29_ClN_2_NaO_5_[M+Na]^+^: 483.1662; found: 483.1661.

*Boc-Gpn-Phe-OH* ***12b***

mp: 80-81 °C; IR (KBr, cm^-1^) : 3364, 2929, 1716, 1652, 1541; ^1^H-NMR (300 MHz, CDCl_3)_ *δ* = 7.42 (*d*, 1H, *J *= 8.7 Hz, Phe NH); 7.12-7.26 (*m*, 5H, H-Ar); 6.68 (*d*, 1H, *J *= 8.7 Hz, Gpn NH); 5.11-5.13 (*m*, 1H, C^α^H of Phe); 3.07-3.13 (*m*, 2H, C^γ^H of Gpn); 2.48-2.55 (*dd*, 1H, J = 14.05, 7.9 Hz, Phe diastereotopic C^β^H); 1.94-2.20 (*m*, 3H, Phe diastereotopic C^β^H and C^α^H of Gpn); 1.52 (*s*, 9H, Boc-CH_3)_; 1.24-1.58 (*m*, 10H, cyclohexyl ring protons) ppm. ^13^C-NMR (75 MHz, CDCl_3)_ *δ* = 176.9 (C of COOH); 170.4 (C of NCO); 158.9 (C of NCOO); 136.4 (C_ipso _of phenyl ring attached to CH_2_); 129.5 (*m*-C^’^s of phenyl ring); 128.3 (*o*-C^’^s of phenyl ring); 126.9 (*p*-C of phenyl ring); 81.7 (C of Boc); 54.5 (*α*-C of Phe); 51.9, 46.7, 42.0, 38.8, 37.7, 37.4, 34.4 (C- Aliphatic); 28.3 (C of Boc-CH_3)_; 26.1, 21.5 ppm. HRMS-ESI: Calcd. for C_23_H_35_N_2_O_5_ [M+1]^+^:419.2543; found: 419.2543. Calcd. for C_23_H_34_N_2_NaO_5_ [M+Na]^+^:441.2363; found: 441.2362. Calcd. for C_23_H_34_KN_2_O_5_ [M+K]^+^:457.2102; found: 457.2102.

*Boc-Baclofen-Phe-Phe-OMe* ***13a***

mp:181-182 °C; IR (KBr, cm^-1^) :3370, 3350, 2924, 2854, 1742, 1681, 1647. ^1^H-NMR (300 MHz, DMSO-*d*_6__)_ Mixture of two diasteromers *δ* = 8.33-8.43 (*m*, 1H, Phe (1) NH); 7.96-8.03 (*m*, 1H, Phe(2) NH); 6.98-7.29 (*m*, 14H, H-Ar); 6.68-6.80 (2*t*, 1H, Baclofen NH); 4.39-4.49 (*m*, 2H, C^α^H of Phe); 3.5 (*s*, 3H, -OCH_3)_; 2 59-3.11 (*m*, 7H, C^β^H of Baclofen, C^γ^H of Baclofen, Phe diastereotopic C^β^H); 2.22-2.39 (*m*, 2H, Baclofen diastereotopic C^α^H); 1.3 (*s*, 9H, Boc-CH_3)_ ppm. ^13^C-NMR (75 MHz, DMSO-*d*_6__)_ *δ* = 171.7, 171.6 (C of COOMe); 171.3 (C of NCO); 170.2, 170.1(C of NCO); 155.5, 155.4 (C of NCOO); 141.4-141.2 (C_ipso_- Cl); 137.8, 137.5, 137.0, 136.9 (C_ipso_ of Phe phenyl ring attached to CH_2_); 130.7 (C_ipso_ of Baclofen phenyl ring attached to CH_2_); 129.5, 129.2, 129.0, 128.9, 128.0, 128.0, 127.9 (*o* &* m*-C^’^s of phenyl rings); 126.6, 126.2, 126.0 (*p*-C of phenyl rings); 77.5 (C of Boc); 53.6, 53.1 (*α*-C of Phe); 51.8 (C of -OCH_3)_; 45.0 (*β*-C of Baclofen); 44.6, 41.4 (*β*-C of Phe); 38.5 (*γ*-C of Baclofen); 36.6 (*α*-C of Baclofen); 28.2 (C of Boc-CH_3)_ ppm. HRMS-ESI: Calcd. for C_34_H_41_ClN_3_O_6_ [M+H]^+^:622.2642; found: 622.2644. Calcd. for C_34_H_40_ClN_3_NaO_6_ [M+Na]^+^:644.2501; found: 644.2500. Calcd. For C_34_H_40_ClKN_3_O_6_ [M+K]^+^:660.2242; found: 660.2241.

*Boc-Gpn-Phe-Phe-OMe* ***13b***

mp: 70-71 °C; IR (KBr, cm^-1^): 3297, 3065, 3012, 1745, 1645, 1690. ^1^H-NMR (300 MHz, CDCl_3)_* δ* = 7.74 (*d*, 1H, *J *= 7.3 Hz, Phe(1) NH); 7.02-7.26 (*m*, 10H, H-Ar); 6.95 (*d*,1H, *J *= 7.5 Hz, Phe(2) NH); 5.1 (*brt*, 1H, Gpn NH); 4.7-4.81 (*m*, 2H, C^α^H of Phe); 3.65(*s*, 3H, -OCH_3)_; 2.89-3.2 (*m*, 4H, Phe diastereotopic C^β^H); 2.81-2.83 (*m*, 2H, C^γ^H of Gpn); 2.0-2.02 (*s*, 2H, C^α^H of Gpn); 1.44 (*s*, 9H, Boc-CH_3)_; 1.0-1.42 (*m*, 10H, cyclohexyl ring protons) ppm. ^13^C-NMR (75 MHz, CDCl_3)_* δ* = 171.8 (C of COOMe); 171.5 (C of NCO); 171.2 (C of NCO); 157.2 (C of NCOO); 137.1, 135.9 (C_ipso_ of phenyl ring attached to CH_2_); 129.3 (*m*-C^’^s of phenyl rings); 128.4 (*o*-C^’^s of phenyl rings); 127.0, 126.7 (*p*-C of phenyl rings); 79.5 (C of Boc); 54.6 (*α*-C of Phe); 53.4 (*α*-C of Phe); 52.1, 52.3 (C of -OCH_3)_; 46.6 (C of CH_2_NH_2)_; 42.3 (*β*-C of Gpn); 37.9 (*β*-C of Phe); 37.2 (*β*-C of Phe); 37.1(C of CH_2_COO); 33.9 (*γ*-C’s of Gpn); 28.4 (C of Boc-CH_3)_; 25.9 (*ω*-C of Gpn); 21.4 (*δ*-C’s of Gpn) ppm. HRMS-ESI: Calcd. for C_33_H_46_N_3_O_6_ [M+H]^+^: 580.3383; found: 580.3383. Calcd. for C_33_H_45_N_3_NaO_6_ [M+Na]^+^: 602.3203; found: 602.3202. Calcd. For C_33_H_45_KN_3_O_6 _[M+K]^+ ^: 618.2945; found: 618.2944.

*Boc-Gpn-Phe-Phe-OH*

mp: 107-108 °C; IR (KBr, cm^-1^) :3380, 2937,1860, 1665, 1636. ^1^H-NMR (300 MHz, DMSO-*d*_6)_ *δ* = 12.5 (*brs*, 1H, -COOH); 8.27 (*d*, 1H, *J *= 8.3 Hz, Phe(1) NH); 8.13 (*d*, 1H, *J *= 7.7 Hz, Phe (2) NH); 7.13-7.28 (*m*, 10H, H-Ar); 6.58 (*brt*, 1H, Gpn NH); 4.57-4.64 (*m*, 1H, C^α^H of Phe (1); 4.40-4.47 (*m*, 1H, C^α^H of Phe); 2.61-3.09 (*m*, 6H, Phe diastereotopic C^β^H, and C^γ^H of Gpn); 1.86-1.97 (*m*, 2H, C^α^H of Gpn); 1.37 (*s*, 9H, Boc-CH_3)_; 0.92-1.27 (*m*, 10H, cyclohexyl ring protons) ppm. ^13^C-NMR (75 MHz, DMSO-*d*_6__)_ *δ* = 172.7 (C of COOH); 171.5 (C of NCO); 170.4 (C of NCO); 156.1 (C of NCOO); 137.8, 137.3 (C_ipso_ of phenyl ring attached to CH_2_); 129.1 (*m*-C^’^s of phenyl rings); 128.2, 127.9 (*o*-C^’^s of phenyl rings); 126.4,126.1 (*p*-C of phenyl rings); 77.5 (C of Boc); 53.4 (*α*-C of Phe); 46.6 (C of CH_2_NH_2)_; 42.3 (*β*-C of Gpn); 37.4, 36.7 (*β*-C of Phe); 36.6 (C of CH_2_COO); 33.2, 32.7 (*γ*-C’s of Gpn); 28.2 (C of Boc-CH_3)_; 25.6 (*ω*-C of Gpn); 21.0 (*δ*-C’s of Gpn) ppm. HRMS-ESI: Calcd. for C_32_H_44_N_3_O_6_ [M+H]^+^:566.3227; found: 566.3227. Calcd. for C_32_H_43_N_3_NaO_6 _[M+Na]^+^: 588.3047; found: 588.3047. Calcd. for C_32_H_43_KN_3_O_6_ [M+K]^+^:604.2795; found: 604.2794.

*Boc-Baclofen-Phe-Phe-OH*

mp: 260-262°C (dec.); IR (KBr, cm^-1^) :3350, 3305, 3029, 1714, 1683, 1648. ^1^H-NMR (300 MHz, DMSO-*d*_6__)_ *δ* = 12.64 (*brs*, 1H, -COOH); 8.22-8.25 (*m*, 1H, Phe (1) NH); 7.94-7.97 (*m*, 1H, Phe(2) NH); 6.99-7.28 (*m*, 14H, H-Ar); 6.67-6.79 (*m*, 1H, Baclofen NH); 4.38-4.45 (*m*, 2H, C^α^H of Phe); 3.02-3.13 (*m*, 1H, C^β^H of Baclofen); 2.93-3.01 (*m*, 2H, C^γ^H of Baclofen); 2.49-2.93 (*m*, 4H, Phe diastereotopic C^β^H); 2.21-2.41 (*m*, 2H, Baclofen diastereotopic C^α^H); 1.30 (*s*, 9H, Boc-CH_3)_ ppm. ^13^C-NMR (75 MHz, DMSO-*d*_6__)_ *δ* = 172.6 (C of COOH); 171.2 (C of NCO); 170.1 (C of NCO); 155.5 (C of NCOO); 141.4, 141.2 (C_ipso_-Cl); 137.9, 137.6, 137.4, 137.3 (C_ipso_ of Phe phenyl ring attached to CH_2_); 130.7 (C_ipso_ of Baclofen phenyl ring attached to CH_2_); 129.4, 129.2, 129.1, 129.0, 128.2, 128.0, 127.9, 127.8 (*m & o*-C^’^s of phenyl rings); 126.4, 126.1, 126.0 (*p*-C of phenyl rings); 77.5 (C of Boc); 53.4 (*α*-C of Phe); 45.1 (*β*-C of Baclofen); 41.4, 38.7 (*β*-C of Phe); 37.3 (*γ*-C of Baclofen); 36.7 (*α*-C of Baclofen); 28.2 (C of Boc-CH_3)_ ppm. HRMS-ESI: Calcd. for C_33_H_39_ClN_3_O_6_ [M+H]^+^:608.2529; found: 608.2528. Calcd. for C_33_H_38_ClN_3_NaO_6_ [M+Na]^+^:630.2349; found: 630.2348.

*H-Baclofen-Phe-Phe-OH* ***14a***

mp: 255-257 °C (dec.); IR (KBr, cm^-1^) :3400, 3300, 3028, 1671, 1646. HRMS-ESI: Calcd. for C_28_H_31_ClN_3_O_4_ [M+H]^+^:508.1998; found: 508.1998. Calcd. for C_28_H_30_ClN_3_NaO_4 _[M+Na]^+^: 530.1820; found: 530.1819. Calcd. for C_28_H_30_ClKN_3_O_4_ [M+K]^+^: 546.1560; found: 546.1559.

*H-Gpn-Phe-Phe-OH* ***14b***

mp: 245-246 °C; IR (KBr, cm^-1^): 3000- 3500, 1590- 1670. ^1^H-NMR (300 MHz, DMSO-*d*_6__)_*δ* = 8.91-8.93 (*d*, 1H, *J *= 7.56 Hz, Phe(1) NH); 7.68-7.7 (*d*, 1H, *J *= 6.9 Hz, Phe (2) NH); 7.0-7.25 (*m*, 10H, H-Ar); 4.36-4.43 (*m*, 1H, C^α^H of Phe(1)); 4.14-4.18 (*m*, 1H, C^α^H of Phe); 2.28-3.15 (*m*, 6H, Phe diastereotopic C^β^H and C^γ^H of Gpn); 2.16-2.27 (*m*, 2H, C^α^H of Gpn); 1.21-1.39 (*m*, 10H, cyclohexyl ring protons) ppm. ^13^C-NMR (75 MHz, DMSO-*d*_6__)_ *δ* = 174.2 (C of COOH); 170.9 (C of NCO); 170.3 (C of NCO); 138.9, 138.2, 129.5, 128.9, 128.2, 128.0, 127.7, 126.1, 125.7(C-Ar) ; 55.4, 54.9 (α-C of Phe); 45.0, 37.7, 37.3, 36.1, 35.0, 32.5 (H-Aliphatic); 25.3 (*ω*-C of Gpn); 22.4 (*δ*-C’s of Gpn) ppm. HRMS-ESI: Calcd. for C_27_H_36_N_3_O_6_ [M+H]^+^:466.27005; found: 466.27014. Calcd. for C_27_H_35_N_3_NaO_6 _[M+Na]^+^: 488.2518; found: 488.2521.

*Boc-Baclofen-Baclofen-OMe* ***15***

mp: 133-135 °C; IR (KBr, cm^-1^): 3352, 3311, 2972, 1735, 1678, 1630. ^1^H-NMR (300 MHz, CDCl_3_) *δ* = 7.24-7.28 (*m*, 4H, *m*-H^’^s of Baclofen Phenyl rings protons); 7.02-7.07 (*m*, 4H, *o*-H^’^s of Baclofen Phenyl rings protons); 6.00-6.16 (*brt*, 1H, Baclofen (2) NH); 4.50-4.55 (*m*, 1H, Baclofen(1) NH); 3.57, 3.58 (*s*, 3H, -OCH_3)_; 3.57-3.61 (*m*, 1H, C^β^H of Baclofen(1); 3.39-3.44 (*m*, 1H, C^β^H of Baclofen(2)); 3.2-3.28 (*m*, 4H, C^γ^H of Baclofen); 2.46-2.52 (m, 3H, Baclofen diastereotopic C^α^H);2.23-2.30 (*dd*, 1H, *J* = 14.1, 6.7 Hz, Baclofen diastereotopic C^α^H); 1.39 (*s*, 9H, Boc-CH_3)_ ppm. ^13^C-NMR (75 MHz, CDCl_3)_ *δ* = 172.1, 172.0 (C of COOMe); 171.1, 171.0 (C of NCO); 156.3 (C of NCOO); 140.0, 139.5 (C_ipso_-Cl); 132.9, 132.8 (C_ipso _of phenyl ring attached to CH_2_); 128.9 (*m*-C^’^s of phenyl rings); 128.8 (*o*-C^’^s of phenyl rings); 79.7 (C of Boc); 51.8, 51.6 (C of -OCH_3_); 45.0, 44.9 (*β*-C of Baclofen (2); 44.2, 44.1 (*β*-C of Baclofen (1); 42.4, 42.3 (*γ*-C of Baclofen (1); 41.3, 41.1 (*γ*-C of Baclofen (2), 41.2; 40.0 (*α*-C of Baclofen (2); 38.2, 37.9 (*α*-C of Baclofen (1); 28.3 (C of Boc-CH_3_) ppm. HRMS-ESI: Calcd. for C_26_H_33_Cl_2_N_2_O_5_[M+H]^+^: 523.1763; found: 523.1763. Calcd. for C_26_H_32_Cl_2_N_2_NaO_5_[M+Na]^+^: 545.1583; found: 545.1582. Calcd. for C_26_H_32_Cl_2_KN_2_O_5_ [M+K]^+^: 561.1320; found: 561.1320.

*Boc-* *Baclofen-Baclofen-OH*

mp: 130-132 °C; IR (KBr, cm^-1^): 3345, 3334, 2981, 1706, 1680, 1645. ^1^H-NMR (300 MHz, DMSO-*d*_6__)_ Mixture of stereoisomers: *δ* = 12.18 (*brs*, 1H, -COOH); 7.76 (*m*, 1H, Baclofen (2) NH); 7.04-7.39 (*m*, 8H, H-Ar); 6.76-6.84 (*m*, 1H, Baclofen(1) NH); 3.54-3.63 (*m*, 1H, C^β^H of Baclofen(2)); 3.02-3.19 (*m*, 5H, C^β^H of Baclofen (1) and C^γ^H of Baclofen); 2.62-2.78 (*m*, 2H, Baclofen diastereotopic C^α^H); 2.22-2.39 (*m*, 2H, Baclofen diastereotopic C^α^H); 1.31 (*s*, 9H, Boc-CH_3)_ ppm. ^13^C-NMR (75 MHz, DMSO-*d*_6__)_ *δ* = 175.8,174.5 (C of COOH); 172.8, 172.7, 170.5, 170.4 (C of NCO); 155.5 (C of NCOO); 141.8, 141.3, 141.2, 141.1 131.1, 130.0, 130.9 129.7, 129.5, 128.9 128.4, 128.0 (C-Ar); 72.4 (C of Boc); 48.4 45.2 42.7 41.3, 41.7 40.7, 40.3 37.6 (C-Aliphatic); 28.2 (C of Boc-CH_3)_ ppm. HRMS-ESI: Calcd. for C_25_H_31_Cl_2_N_2_O_5_ [M+H]^+^: 509.16096; found: 509.16088. Calcd. For C_25_H_30_^35^Cl_2_N_2_NaO_5_ [M+Na]^+^: 531.1429; found: 531.1428. Calcd. for C_25_H_30 _^35^Cl_2_KN_2_O_5 _ [M+K]^+ ^: 547.1170; found: 547.1169.

*Boc-Baclofen-Baclofen-Phe-Phe-OMe* ***16***

mp: 128-129 °C; IR (KBr, cm^-1^) :3306, 1700, 1689, 1642.

HRMS-ESI: Calcd. for C_44_H_51_Cl_2_N_4_O_7_ [M+H]^+^: 817.3143; found: 817.3141. Calcd. for C_44_H_50_Cl_2_N_4_NaO_7_ [M+Na]^+^: 839.2955; found: 839.2954. Calcd. for C_44_H_50_Cl_2_KN_4_O_7_ [M+K]^+^: 855.2704; found: 55.2702. 

*Boc-Baclofen-Baclofen-Phe-Phe-OH* ***17*** *: *mp: 121-123 °C, IR (KBr, cm^-1^): 3100-3300, 1714, 1658. 

HRMS-ESI: Calcd. for C_43_H_49_Cl_2_N_4_O_7_ [M+H]^+^: 803.2985; found: 803.2984 ; Calcd. for C_43_H_48_Cl_2_N_4_NaO_7_ [M+Na]^+^: 825.2810; found: 825.2809.

*H-Baclofen-Baclofen-Phe-Phe-OH 18:*

mp: 110-112 °C; IR (KBr, cm^-1^): 3200-3400, 1678.

HRMS-ESI: Calcd. for C_38_H_41_Cl_2_N_4_O_5_ [M+H]^+^: 703.2449; found: 703.2449. Calcd. for C_38_H_40_Cl_2_N_4_NaO_5_ [M+Na]^+^: 725.2272; found: 25.2271.

## Results and Discussion

**Scheme 1:**
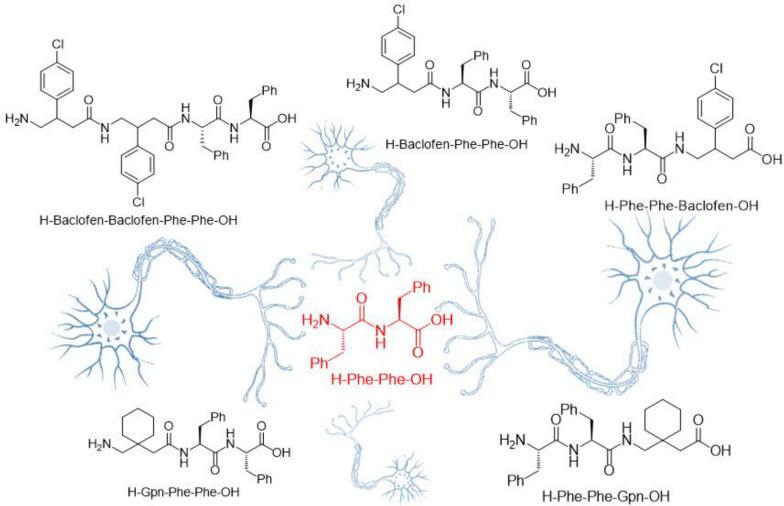
Structure of the used aminoacids

To improve the functionality of F-F dipeptide, two γ-aminobutyric acids, Gpn, and Baclofen (Scheme 1) were introduced to its structure to increase the π-π stacking, and thus lipophilicity properties. Gabapentin is an anticonvulsant medication and its properties is owing to the presence of lipophilic cyclohexyl in its structure penetrating into the blood-brain barrier and central nervous system (26). Besides, Baclofen includes 4-chlorophenyl ring which its presence in tri-peptide structure may increase the π-π stacking. 

To synthesize tripeptides, two strategies were employed. The first one was relied on the core of Phe-Phe to introduce the third amino acid. The second one, Phenylalanine was bound to the C-terminus of γ-aminobutyric acids. In both strategies, solution phase peptide synthesis strategy was used (27). 

**Scheme 2:**
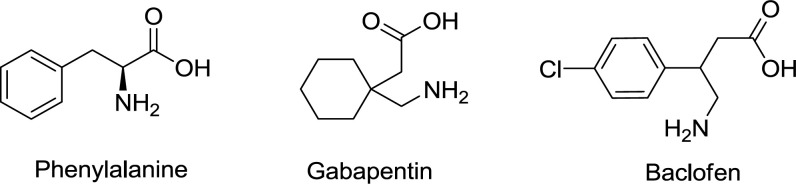
Synthesis of tri-peptide starting from Phenylalanine; Ar: 4-ClC_6_H_4_, Reagents and conditions: (a) EtOAc, H–Phe–OMe, TBTU, HOBt, DIEA, R. T.; (b) MeOH, 2 M NaOH, H_2_O; (c) EtOAc, H–Gpn–OMe, TBTU, HOBt, DIEA, R. T.; (d) EtOAc, H–Baclofen–OMe, TBTU, HOBt, DIEA, R. T.; (e) HSiEt_3_, TFA/CH_2_Cl_2_

To start the first strategy, protection reactions were essential for the preparation of amino acids, which include: carboxylic acid protection of Boc-Phe-OH through a methylation process using thionyl chloride in MeOH, and amine protection of Phe by di-*tert*-butyl dicarbonate (Boc_2_O). As shown in Scheme 2, the reaction steps involved: a) coupling reaction of protected amino acids which are Boc-Phe-OH and H-Phe-OMe using TBTU/HOBT as the coupling reagent, b) basic hydrolysis of methyl ester using NaOH in MeOH. c and d) Coupling of Boc-Phe-Phe-OH with methyl ester protected γ-aminobutyric acids (H_2_N-Gpn-OMe and H_2_N-Baclofen-OMe) and then, deprotection of ester and amine, respectively. The final products of this strategy were H-Phe-Phe-Gpn-OH and H-Phe-Phe-Baclofen-OH. 

It is notable to mention that coupling of Boc-Phe-OH with phenylalanine methyl ester H-Phe-OMe was accomplished through the standard epimerization-free condensation reaction (HOBt) according to Ley’s protocol (28). Moreover, treatment of the protected tripeptide with a triethyl silane and a mixture of CH_2_Cl_2_/TFA (1:1 ratio) lead to the deprotection of its terminal amino group producing the unprotected tripeptide

**Scheme 3:**
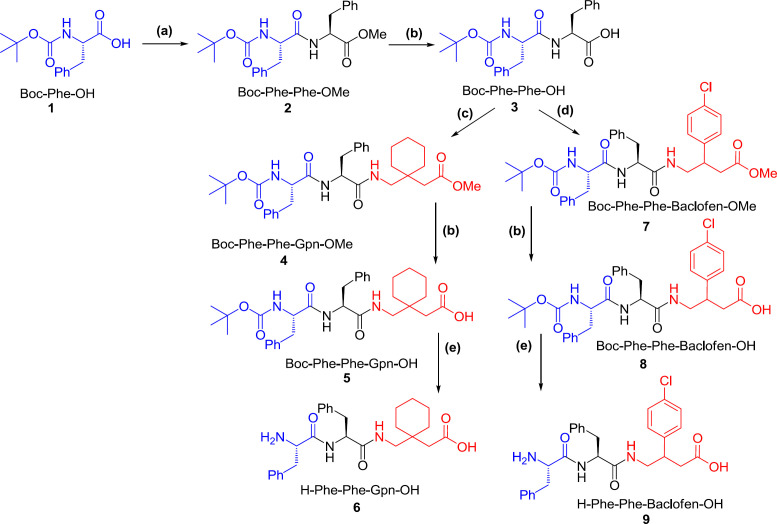
Synthesis of tri-peptide starting from γ-aminobutyric acids (a) EtOAc, H–Phe–OMe, TBTU, HOBt, DIEA, R. T.; (b) MeOH, 2 M NaOH, H_2_O; (c) HSiEt_3_, TFA/CH_2_Cl_2_

**Scheme 4:**
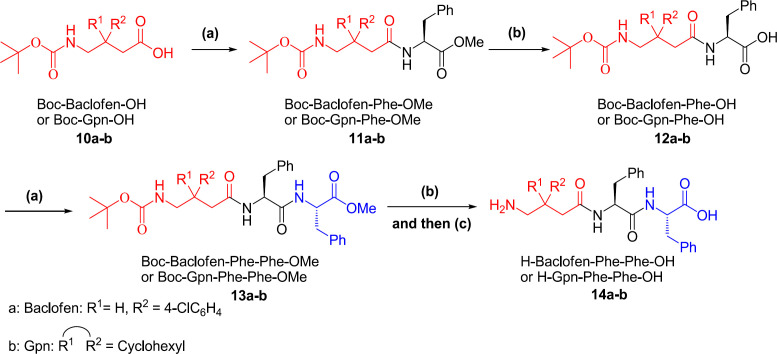
Synthesis of *tetra*-peptide starting from Baclofen (a) EtOAc, H–Baclofen–OMe, TBTU, HOBt, DIEA, R.T.; (b) MeOH, 2 M NaOH, H_2_O; (c) EtOAc, H–Phe–Phe-OMe, TBTU, HOBt, DIEA, R. T.; (d) HSiEt_3_, TFA/CH_2_Cl_2_

Synthesis of tripeptides H-Gpn-Phe-Phe-OH and H-Baclofen-Phe-Phe-OH was started from their corresponding γ-aminobutyric acid (Scheme 3). In this regard, amino group of γ-aminobutyric acid was initially protected using Boc_2_O reagent and then treated with ester of Phe. After cleavage of the ester group of the formed dipeptide, previous reaction was repeated followed by deprotection of acid and amine groups to obtain tripeptides H-Baclofen-Phe-Phe-OH and H-Gpn-Phe-Phe-OH. The process of synthesizing tetrapeptide H-Gpn-phe-phe-OH involved the same processes with a difference that the first step included reaction of Boc-Baclofen-OH with H-Baclofen-OMe (Scheme 4).

In order to make sure the successfulness of each reaction steps, the products were analyzed by FT-IR and ^1^H and ^13^C NMR (see supplementary data). In ^1^H NMR spectra of H–Phe–OMe, the appearance of a sharp peak at 3.62 ppm, corresponding the methyl group, proved the formation of ester. 

**Figure 2:**
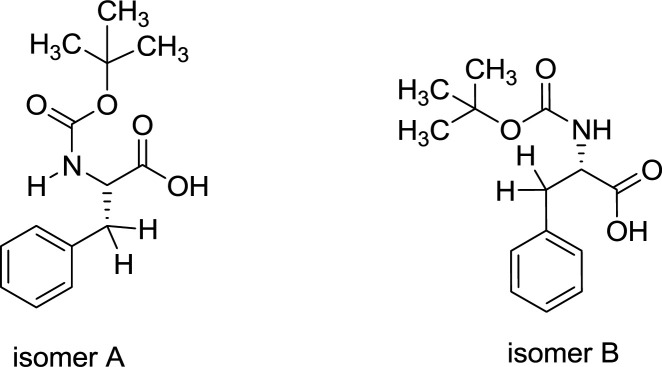
Isomerization of the amide bond in Boc-Phe-OH

Due to the twofold peaks in both ^1^H and ^13^C NMR spectra of Boc-Phe-OH, it was found that this product constitutes two isomers with the ratio of 64:36, this is as a result of amide resonance by the partial shift of NH proton to the carbonyl group (Figure 2). Isomer A is the major one and in isomer B the chemical shift for the -NH group is 6.59 ppm that is related to the intramolecular hydrogen bonding between -NH and -C= O group (29). 

Observing the peak of *t*-Bu group’s hydrogens in ^1^H NMR and the peaks of urethane carbonyl and carbon atoms of *t*-Bu group were the proof for the Boc-protection of Baclofen and Gpn. To confirm the formation of peptide bonds, H-H COSY 2D NMR was also considered in addition to the conventional analyses. This spectrum helped to find the H-C_α_ bonded to the NH. ESI Mass spectrometry detected the mass of each synthesized peptide. 

Since self-assembled peptide-based hydrogels have brought better interaction, and the fact that γ-amino acid including peptide may form gel, the synthesized small peptides are under investigation for the formation of gel and self-assembled peptides.

## Conclusion

In conclusion, the synthesis of novel series of tri- and tetrapeptides were described and confirmed by inserting *γ*-amino acids of Gpn and Baclofen into the structure of Phe-Phe dipeptide. Adding the bioactive γ-amino acids to the Phe-Phe sequence could affect the lipophilicity and self-assembly of the peptides. The increased lipophilic properties of tri- and tetrapeptides leading to the nanostructural formation may affect their permeability onto the nervous system. The research to find the gel formation condition and also their activity is in progress in our lab. 

## References

[1] Cordingley MG, Register RB, Callahan P, Garsky VM, Colonno R (1989). Cleavage of small peptides in-vitro by human rhinovirus 14 3C protease expressed in Escherichia coli. J. Virol..

[2] Mackenzie B, Fei Y-J, Ganapathy V, Leibach FH (1996). The human intestinal H+/oligopeptide cotransporter hPEPT1 transports differently-charged dipeptides with identical electrogenic properties. Biochim. Biophys. Acta.

[3] Boldyrev AA, Severin SE (1990). The histidine-containing dipeptides, carnosine and anserine: distribution, properties and biological significance. Adv. Enzyme Regul..

[4] Reches M, Gazit E (2003). Casting metal nanowires within discrete self-assembled peptide nanotubes. Science.

[5] Skaat H, Chen R, Grinberg I, Margel S (2012). Engineered polymer nanoparticles containing hydrophobic dipeptide for inhibition of amyloid-β fibrillation. Biomacromolecules.

[6] Gazit E (2002). A possible role for π-stacking in the self-assembly of amyloid fibrils. FASEB J..

[7] Ji W, Yuan C, Zilberzwige-Tal S, Xing R, Chakraborty P, Tao K, Gilead S, Yan X, Gazit E (2019). Metal-ion modulated structural transformation of amyloid-like dipeptide supramolecular self-assembly. ACS Nano.

[8] James III WH, Müller CW, Buchanan EG, Nix MGF, Guo L, Roskop L, Gordon MS, Slipchenko LV, Gellman SH, Zwier TS (2009). Intramolecular amide stacking and its competition with hydrogen bonding in a small foldamer. J. Am. Chem. Soc..

[9] Horne WS, Boersma MD, Windsor MA, Gellman SH (2008). Sequence-Based Design of α/β-Peptide Foldamers That Mimic BH3 Domains. Angew, Chem. Int. Ed..

[10] Nagy A, Gőz VG, Pintér I, Farkas V, Perczel A (2019). α/β-Chimera peptide synthesis with cyclic β-sugar amino acids: the efficient coupling protocol. Amino acids.

[11] Au C, Gonzalez C, Leung YC, Leung YC, Mansour F, Trinh J, Wang Z, Hu X-G, Griffith R, Pasquier E, Hunter L (2019). Tuning the properties of a cyclic RGD-containing tetrapeptide through backbone fluorination. Org. Biomol. Chem..

[12] Yamada K, Matsumoto R, Suzuki Y, Mori S, Kitajima S (2020). Design, synthesis and evaluation of unnatural peptides as T1R2/T1R3 PAMs. Bioorg. Med. Chem. Lett..

[13] Sarkar B, Mahapa A, Chatterji D, Jayaraman N (2019). Sugar vinyl sulfoxide glycoconjugation of peptides and lysozyme: Abrogation of proteolysis at the lysine sites. Biochemistry.

[14] Schöwe MJ, Keiper O, Unverzagt C, Wittmann V (2019). A tripeptide approach to the solid-phase synthesis of peptide thioacids and N-glycopeptides. Chem. Eur. J..

[15] Sršan L, Ziegler T (2020). Synthesis of new asparagine-based glycopeptides for future scanning tunneling microscopy investigations. Beilstein J. Org. Chem..

[16] Burnsed JC, Heinan K, Letzkus L, Zanelli S (2020). Gabapentin for pain, movement disorders, and irritability in neonates and infants. Dev. Med. Child Neurol..

[17] Vasudev PG, Chatterjee S, Shamala N, Balaram P (2009). Gabapentin: a stereochemically constrained γ-amino acid residue in hybrid peptide design. Acc. Chem. Res..

[18] Quijano RR, Leu RM-Y (2018). 1154 Baclofen therapy for sleep-related symptoms in a child diagnosed with a newly described rare genetic syndrome: a case report. Sleep.

[19] Dhingra AK, Chopra B, Dass R (2019). Prodrug approach: An alternative to improve pharmacokinetic properties. Int. J. Bioorg. Chem..

[20] Katuri JV, Nagarajan K (2019). Hofmann rearrangement of primary carboxamides and cyclic imides using DCDMH and application to the synthesis of gabapentin and its potential peptide prodrugs. Tetrahedron Lett..

[21] Wan Y, Baltaze J-P, Kouklovsky C, Miclet E, Alezra V (2019). Unexpected dimerization of a tripeptide comprising a β,γ-diamino acid. J. Pept. Sci..

[22] Wan Y, Auberger N, Thétiot-Laurent S, Bouillère F, Zulauf A, He J, Courtiol-Legourd S, Guillot R, Kouklovsky C, Cote des Combes S, Pacaud C, Devillers I, Alezra V (2018). Constrained cyclic β, γ-diamino acids from glutamic acid: synthesis of both diastereomers and unexpected kinetic resolution. Eur. J. Org. Chem..

[23] Thétiot-Laurent S, Bouillère F, Baltaze J-P, Brisset F, Feytens D, Kouklovsky C, Miclet E, Valérie Alezra V (2012). Original β, γ-diamino acid as an inducer of a γ-turn mimic in short peptides. Org. Biomol. Chem..

[24] Awada H, Grison CM, Charnay-Pouget F, Baltaze J-P, Brisset F, Guillot R, Robin S, Hachem A, Jaber N, Naoufal D, Yazbeck O, Aitken DJ (2017). Conformational Effects through Hydrogen Bonding in a Constrained γ-Peptide Template: From Intraresidue Seven-Membered Rings to a Gel-Forming Sheet Structure. J. Org. Chem..

[25] Melchionna M E, Styan K, Marchesan S (2016). The Unexpected Advantages of Using D-Amino Acids for Peptide Self- Assembly into Nanostructured Hydrogels for Medicine. Curr. Top. Med. Chem..

[26] Goa KL, Sorkin EM (1993). Gabapentin. Drugs.

[27] Lloyd-Williams P, Albericio F, Giralt E (1997). Chemical approaches to the synthesis of peptides and proteins.

[28] Baxendale IR, Ley SV, Smith CD, Tranmer GK (2006). A flow reactor process for the synthesis of peptides utilizing immobilized reagents, scavengers and catch and release protocols. Chem. Commun..

[29] Günther H (2013). NMR spectroscopy: basic principles, concepts and applications in chemistry.

